# What do hypnotics cost hospitals and healthcare?

**DOI:** 10.12688/f1000research.11328.2

**Published:** 2017-06-28

**Authors:** Daniel F. Kripke

**Affiliations:** 1 University of California San Diego, La Jolla, CA, 92037-2226, USA

**Keywords:** hospital costs, health care costs, hypnotics and sedatives, infection, depressive disorders, overdose, mortality, epidemiology

## Abstract

Hypnotics (sleeping pills) are prescribed widely, but the economic costs of the harm they have caused have been largely unrecognized. Randomized clinical trials have observed that hypnotics increase the incidence of infections. Likewise, hypnotics increase the incidence of major depression and cause emergency admissions for overdoses and deaths.  Epidemiologically, hypnotic use is associated with cancer, falls, automobile accidents, and markedly increased overall mortality.  This article considers the costs to hospitals and healthcare payers of hypnotic-induced infections and other severe consequences of hypnotic use. These are a probable cause of excessive hospital admissions, prolonged lengths of stay at increased costs, and increased readmissions. Accurate information is scanty, for in-hospital hypnotic benefits and risks have scarcely been studied -- certainly not the economic costs of inpatient adverse effects.  Healthcare costs of outpatient adverse effects likewise need evaluation. In one example, use of hypnotics among depressed patients was strongly associated with higher healthcare costs and more short-term disability. A best estimate is that U.S. costs of hypnotic harms to healthcare systems are on the order of $55 billion, but conceivably might be as low as $10 billion or as high as $100 billion. More research is needed to more accurately assess unnecessary and excessive hypnotics costs to providers and insurers, as well as financial and health damages to the patients themselves.

## Briefly summarizing the risks of hypnotics

Evidence of hypnotic harms is growing - the American Geriatrics Society has recommended that the popular hypnotic drugs be avoided for older patients, who are almost half of hospital patients ^1^. Similarly, the American College of Physicians (ACP) recommended that cognitive behavioral therapy should be the first-choice treatment for insomnia, and the ACP guideline expressed doubt on whether hypnotics were worth the risks, even as secondary choices for short-term use ^2, 3^. A 2017 American Academy of Sleep Medicine (AASM) guideline reiterated that cognitive-behavioral therapy is the first-choice treatment for insomnia. For pharmacologic treatment of chronic insomnia, AASM gave “WEAK” positive recommendations for use of certain hypnotics, but qualified those recommendations by conceding inadequate evidence concerning the severe risks of hypnotic drugs discussed below, by conceding that their recommendations were limited to insomnia patients without much comorbidity (probably the minority), and by noting that their recommendations might not be applicable to payer perspectives ^4, 5^.

The more severe risks of hypnotic drugs are rarely recognized.

- Randomized controlled trials show:
  a) Hypnotics increase incidence of infections, with a mean 44% increase in controlled trials ^6^. Moreover, infections are associated with depression and suicide ^7^.
  b) Hypnotics increase the incidence of major depression by more than two-fold ^8^.
- Death certificates list hypnotics and other benzodiazepine agonists among causes of as much as 1 out of 3 U.S. opiate overdose deaths and may be present in about half of suicides ^7^.
- Epidemiologic studies demonstrate more risks associated with hypnotics:
  a) In-hospital falls, e.g. over 3 times as many falls have been observed among patients receiving zolpidem ^9^. Outpatient falls are also increased.
  b) Hypnotic use is associated with up to double the motor vehicle crash rate ^10^.
  c) Emergency room visits related to hypnotic ingestions have been increasing ^11^.
  d) Rates of specific cancers, especially lung and esophagus, have multiplied among hypnotic users ^7^.
  e) According to electronic records systems in 5 countries, overall mortality appeared increased 2-fold to 4-fold among patients receiving hypnotics, after adjustments for comorbid risk factors and confounders ^7^.

Hypnotic harms to patients have been documented in more detail elsewhere, with critiques on the strengths and limitations of such evidence ^7, 12^. It is important to recognize that causality of some of these harms has been supported by randomized controlled trials or death certificates, and for other associated harms, demonstrated association may not be sufficient evidence for causality because of confounding comorbidities. Here the focus is on estimating financial harms to health systems even when lacking precise estimates of the hypnotics-caused components of these harms.

What has been missing from current documentation is a detailed report on the cost of hypnotics to hospitals, insurers, and managed care, where the minimal benefits are weighed against the severe harms. Factual economic data have been so sparse that we must use fragmentary evidence and some speculation to estimate how much hypnotics cost the U.S. medical systems. Additional studies are needed before precise cost estimates can be made.

## The benefits of hypnotics are trivial or absent, without documented cost savings

An authoritative systematic review biased towards hypnotics, limited to subjective outcomes of outpatient insomnia patients, restricted to published controlled trials, and including studies of greater-than-recommended doses, found low-strength evidence for weak benefits for two “Z” hypnotics used mainly in doses higher than recommended ^3, 7^. Insufficient evidence of any benefit was found for the other benzodiazepine agonists ^3^. Moreover, the authors stated that, “it is not known how many minutes’ change in SOL, TST, or WASO indicate clinically meaningful improvement ^3^.” In other words, it is not known if the weak benefits reported were clinically meaningful even at high doses that are considered unsafe. An older definitive review of objective polysomnographic data that included data from unpublished trials concluded that hypnotics produced little or no objective improvements in total sleep in currently-recommended doses and no verified overall health benefits ^7^.

## Available information about the cost of hypnotic harms to hospitals and healthcare

Up to now, medical literature has projected costs of insomnia harms but has hardly mentioned what the harms from treating with hypnotics may cost. The presence of insomnia is obviously confounded by association with prescription of hypnotics, though not as closely as one might expect ^7, 12^. In a study of 55,000,000 managed care patients, only 31% of those receiving hypnotic medication had a diagnosis of insomnia ^13^. The fraction of insomnia patients receiving hypnotics is quite variable depending on the patient samples and definitions of insomnia. Another complication is that using hypnotics may actually cause insomnia ^14^, at least following hypnotic withdrawal. For example, in the long run, CBT-treated patients tapered off zolpidem slept better over time than a contrast group randomly provided with continuing zolpidem ^15^. Consequences of insomnia such as absenteeism, automobile crashes, and increased medical costs were estimated to be costing the U.S. over $15 billion in 1993 ^16^. Several more recent cost estimates have been far higher, as high as $216 billion in 2015 dollars ^17^. These studies generally made little attempt to differentiate costs caused by insomnia itself from costs of confounding comorbidities and correlated hypnotic harms ^18^. Some insomnia cost studies were sponsored by hypnotic manufacturers or others with interests in attributing the costs to insomnia.

Several studies have attributed costs associated with hypnotic prescribing to insomnia, ignoring that less than half of the prescriptions are given to patients with diagnosed insomnia ^13, 19^. Moloney et al. found that due to recent “medicalization” of sleeplessness, from 1993 to 2007, hypnotic prescribing grew much more rapidly than diagnoses of insomnia, so that in physician office visits, hypnotic prescribing grew to over 3 times the rate of sleeplessness-related complaints ^20^. One study used a prescription claim for a hypnotic as an explicit marker for insomnia, in order to compare cohorts with and without insomnia among 87,461 depressed patients. Hypnotic use was associated with more comorbidity-adjusted hospitalization, more frequent ER visits, 12-month healthcare costs that were $3,918 higher, and more short-term disability ^21^. For the authors to attribute these cost correlates of hypnotic prescription claims to insomnia (or underlying depression) and not to the hypnotics themselves seemed illogical. A similarly flawed study of a national sample of insured workers found yearly health costs were $936 higher among those with insomnia, but 2/3 of the insomnia cohort were defined by receiving hypnotics without having received a recorded diagnosis of insomnia ^22^. Another nationwide study found that insomnia was correlated with prolonged hospital stay, but lacked data to determine whether length of stay was more closely correlated with hypnotic use rather than with insomnia diagnoses ^23^. A study of insomnia patients both before and after treatment versus controls found an 85% increase in health costs of insomnia patients treated with sedatives/hypnotics as opposed to insomnia patients that were not treated with hypnotics ^24^. The authors attributed this difference to more serious underlying conditions amongst those treated, without considering the possibility that the treatment itself was increasing costs. A study in Taiwan found that in contrast with a cohort without insomnia that did not use sedatives or hypnotics, a comorbidity-matched cohort with a diagnosis of insomnia suffered more acute myocardial infarction and stroke, but only among those taking hypnotics or other sedatives ^25^. This may suggest that after control for insomnia, it was the sedative/hypnotics causing myocardial infarctions and stroke. Other studies relating insomnia to health care costs have explicitly found greater healthcare costs among those given prescription treatment for insomnia ^26, 27^.

A Mayo Clinic study found that hospital patients who received zolpidem had a 2% longer length-of-stay (not statistically significant), possibly due to their triple hazard of falls ^9^. Although a 2% average increase in length of stay might appear small, such small mean increases would cost billions of dollars if pervasive throughout the United States. Another study found that in-hospital benzodiazepine prescriptions were associated with 23% higher readmissions ^28^. We must recognize that without randomized placebo controls, none of these studies can offer definite proof on whether hypnotics or insomnia cause increased health costs.

It is ironic that several of the studies that document hypnotic prescriptions as being associated with increased healthcare costs were sponsored by hypnotic manufacturers, when they had intended to attribute these costs to insomnia.

## In-hospital hypnotic cost benefits and risks

Fifty years ago, it was routine to prescribe an “as-needed” hypnotic with almost every hospital admission. In 1982, Perry and Wu reviewed 331 charts of a distinguished teaching hospital and reported that, “Most surgical patients (96%) and a large number of medical patients (46%) had hypnotic agents prescribed on admission without a recorded reason, without the patient’s request or knowledge, and without a statement in the medical chart indicating whether the therapeutic objectives were met ^29^.” Personal communications indicate that routine hypnotic prescribing without evidence of benefit is still a common practice in many of the most renowned academic medical centers. The recent Mayo Clinic report is an example that listed only 32% of patients given zolpidem as having an insomnia diagnosis ^9^.

An up-to-date systematic review of 15 in-hospital controlled trials of hypnotic drugs going back to 1983 found that only one of the included trials (of intravenous dexmedetomidine) showed a convincing advantage for sleep efficiency ^30^, even though several of the studies involved such intravenous drugs. Out of the 15 studies, 5 showed some evidence that oral benzodiazepines reduced sleep latency, but most of the treated patients still had abnormal sleep latencies exceeding 30 minutes. The review concluded with “insufficient evidence to suggest that pharmacotherapy improves the quality or quantity of sleep in hospitalized patients suffering from poor sleep ^30^,” and no other health or cost benefits were documented.

The controlled hospital trials were not designed to assess the costs of hypnotic harms. Indeed, I know of no formal studies on the health cost of harms produced by in-hospital administration of hypnotics, whether randomized or not. It is hard to imagine how drugs that are known to increase the incidence of infection and depression and are strongly associated with in-hospital falls could fail to increase hospital costs. 

## Hypnotic risks cannot be justified when given to patients without diagnosed insomnia

As previously mentioned, most prescriptions for hypnotics are given to patients without diagnosed insomnia, even though insomnia is the sole approved indication for most hypnotics. Zolpidem takes up over 70% of the contemporary U.S. hypnotics’ market. Most zolpidem prescriptions have been given to patients who had one or more hazardous contraindications, such as concomitant use of opiates or other sedatives, age over 60, alcoholism, history of depression or use of antidepressants ^19, 31, 32^. Most outpatient hypnotic prescriptions have been renewals beyond recommended durations at above-recommended doses ^19, 32, 33^. This lack of indication or documented benefit is characteristic of hypnotic prescribing, and it is hard to understand what could justify the risks and costs of supplying the benzodiazepine-agonist hypnotics.

## Cost estimates for specific hypnotic harms

Excessive mortality may be the most expensive harm caused by hypnotics. It is possible to loosely estimate the related costs. The 2006–2008 estimate from the Geisinger Health Study supplement indicated that hypnotics cause roughly 18% of all adult deaths ^34^. Considering that about 27% of Medicare costs (U.S. government payments for healthcare of people aged mainly over 65) are incurred in the last year of life, mainly shortly before death, the costs of hypnotics to Medicare in 2015 caused by increased mortality rates could be roughly $31 billion: 0.18 × 0.27 × $646.2 billion ^35^. Current U.S. hypnotic prescriptions may be about as frequent as they were in 2006–2008, but current Medicare expenditures in 2017 would be a bit higher. Not all Medicare expenditures in the year before death would be related to the damage cause by hypnotics, but there would be counterbalancing hypnotic-related expenditures for patients before the year of death. Moreover, a substantial portion of the medical costs would have fallen on payers other than Medicare such as Medicaid, a government health provider for all-age indigent people costing $545.1 billion ^35^. A somewhat different estimate of total population last-year-of-life care for 2011 was $205 billion ^36^, of which 18% would be $36.9 billion. The number of deaths statistically associated with hypnotic use may greatly overestimate the deaths attributable causally to hypnotics, but likewise the attributable deaths may be underestimated ^12^. A $31 billion yearly Medicare cost estimate is quite possibly inaccurate, but it represents, in my opinion, a reasonable estimate of the cost magnitude for hypnotic-caused mortality, based on current evidence.

The costs of hypnotic-induced infections cannot be accurately estimated. We can gain a perspective on hospital infection costs from 2013 data on the U.S. hospital costs for treatment of septicemia and pneumonia alone, which together were estimated as reaching about $33 billion ^37^. Between 5–10% of these costs came from readmissions. Hypnotics have been shown to cause infections ^6^, e.g., benzodiazepines were associated with 54% higher rates of pneumonia ^38^. Of course, not all infections treated in hospitals are caused by hypnotics or arose in-hospital. I might imagine that hypnotic-caused inpatient-treated infections could cost anywhere up to $20 billion per year. Also, hospital-acquired infections might not be reimbursable by Medicare payments but might fall on other funding sources.

Hypnotics increase the incidence of depression ^7^. Estimating that there are about 14.8 million people in the U.S. each year suffering from major depressive disorders, and that 5.8% took hypnotics ^21, 39^, the total medical costs would add up to about $3.4 billion per year for depression attributable to hypnotics, if the added healthcare cost was around $3918 for each person ^21, 39^.

The medical costs of falls among U.S. adults aged 65 or older were estimated to be about $32 billion for 2015 ^40^. Of these costs, around 63% covered hospitalizations, 21% emergency department visits, and 16% outpatient visits. The average cost per fall was about $30,000. Unfortunately, there are no data available that estimate total numbers of outpatient or inpatient falls attributable to hypnotics in the U.S. However in 2010, among patients hospitalized at the Mayo Clinic, I infer from the number of falls among patients who received zolpidem, their adjusted hazard ratio, and the total falls, that 29% of total inpatient falls could be attributable to zolpidem, although there were only data on 11.8% of patients receiving any zolpidem ^9^. Presumably, the costs of in-hospital falls were not reimbursed by Medicare charges.

Automobile crashes in 2010 were estimated to generate $23 billion in U.S. medical costs ^41^. It is known that people who take sedatives such as zolpidem have higher crash rates. Taken from Washington State health plan data ^10^, sedative users had around twice the crash rate when compared to non-users, after controlling for comorbidities. Nationally, between 3–10% of adults take a hypnotic each year, so we might infer that 3–10% of crashes could be caused by hypnotics, costing roughly $0.6–2.0 billion per year for medical costs. I am inclined to think that the percentage of U.S. adults taking hypnotics in a given year is at the higher end of that estimate, since some studies give even higher estimates ^42, 43^, and press reports suggest current prescriptions are in a range of 40–50 million per year.

National U.S. costs of cancer medical care are projected to reach $158 billion in 2020 ^44^. If we use the Geisinger Health Study ^34^ as a model, $1 to $3 billion of cancer care costs could be associated with hypnotic use each year.

Combining costs of excess mortality, infections, depression, falls, automobile crashes, and cancer, my best estimate is that hypnotics cost hospitals and medical payers somewhere around $55 billion per year, acknowledging an uncertainty range that likely falls between $10 billion and $100 billion. Similarly, assuming that from about 250 million adults in the U.S., 3% to 10% take hypnotics in a given year, and estimating yearly costs related to the hypnotics to range between $936 ^22^ and $3918 ^21, 22^, we can estimate the costs to fall in between $6.3 to $95.4 billion (probably close to the higher figures), consistent with the cumulative cost estimate taken from harm components. Wherever the true costs may fall, within that $10 to $100 billion likely range, these costs are great enough that studies are needed to assess the costs more reliably.

## What more can be learned?

With the recent expansion of electronic medical records, many hospital systems and insurance systems already have sufficient data in their existing electronic records to estimate the outcomes and costs associated with hypnotics prescribing, including hospital admissions, infections, falls, and incident delirium and dementia, lengths of stay, and readmissions. Such available data could give us a much clearer idea of costs associated with in-hospital hypnotic prescribing, but control for comorbidities and other confounders could not assure an accurate estimate of the causal component of associated costs.

Fortunately, it is becoming increasingly possible to utilize genetic data and “Mendelian randomization” to effectively compare groups who received hypnotics due to random genetic propensities with those who did not ^45^. With the increasingly widespread development of personalized medicine, involving genotyping and whole-genome analyses, an increasing number of hospital systems will have accumulated sufficient genetic data to isolate the causal contribution of hypnotics to infection, hospitalization, depression, hospital readmissions, cancer and mortality.

Because of ethical and practical difficulties and manufacturer liability concerns, it appears unlikely that anyone will ever do large enough randomizing hypnotic vs placebo drug trials to demonstrate the costs of hypnotic harms accurately. Fortunately, an alternative randomizing strategy relying on patient choice after education and patient-empowerment has been suggested: such studies might be integrated into the wellness-promotion and cost-reduction programs of managed care organizations ^46^.

## Conclusion

It might be years before more reliable data are assembled on the harm costs and cost-benefits of hypnotics. Meanwhile, hospital leaders and managed care and insurance administrators would be wise to infer from available evidence that the costs of hypnotic harms exceed any cost benefits of hypnotics. A decision to protect patients from hypnotics would also protect payer budgets.
