# Supplementary material for: What do hypnotics cost hospitals and healthcare?
Source: F1000Res. 2017 Jun 28;6:542. Originally published 2017 Apr 21. [Version 2] doi: 10.12688/f1000research.11328.2 (PMC5516216; doi:10.12688/f1000research.11328.2)
Supplement: Peer review history

# Peer review: Referee response for version 2

**DOI:** 10.5256/f1000research.12977.r23862

**Published:** 2017-07-14

**Authors:** Michael A. Grandner

**Affiliations:** 1 Sleep and Health Research Program, Department of Psychiatry, College of Medicine, University of Arizona, Tucson, AZ, USA

I have reviewed the revised version and believe that all issues and concerns have been adequately addressed.

I have read this submission. I believe that I have an appropriate level of expertise to confirm that it is of an acceptable scientific standard.

# Peer review: Referee response for version 2

**DOI:** 10.5256/f1000research.12977.r23863

**Published:** 2017-07-10

**Authors:** Leon C. Lack

**Affiliations:** 1 School of Psychology, Flinders University of South Australia, Adelaide, Australia; 2 Adelaide Institute for Sleep Health, Repatriation General Hospital, Daw Park, Australia

I have studied Version 2 of this article and read the authors comments and am satisfied that it should now be approved for publication.

I have read this submission. I believe that I have an appropriate level of expertise to confirm that it is of an acceptable scientific standard.

# Peer review: Referee response for version 1

**DOI:** 10.5256/f1000research.12228.r23573

**Published:** 2017-06-19

**Authors:** Leon C. Lack

**Affiliations:** 1 School of Psychology, Flinders University of South Australia, Adelaide, Australia; 2 Adelaide Institute for Sleep Health, Repatriation General Hospital, Daw Park, Australia

The author has done considerable work in assembling data relating the use of sedatives and hypnotic drugs to harmful outcomes and increased healthcare costs. The main thrust of this article is, assuming that hypnotic use causes harmful health outcomes, what would be the economic costs of this harm?

 However, the question about hypnotic use by itself causing harm and health morbidity is still frustratingly not completely resolved. The author amasses a wealth of evidence associating the two, lending strength to the notion of causality but not confirming it. There is good experimental evidence showing negative effects of hypnotics including slowed reactions, cognitive and memory impairments, impaired balance arising from their sedating effects both before sleep and, for longer acting hypnotics, after sleep. However, the evidence for the direct effects of hypnotics on physical health measures is not as strong since these are largely correlation studies.

 The greater increase in health morbidity and mortality in those prescribed hypnotics may arise from the possibility that hypnotics are more likely to be prescribed by health professionals in cases with greater and more serious co-morbidity and those likely to have increased mortality risk. This self selection of hypnotic prescriptions to those likely to have higher morbidity and mortality may account for all or some of the subsequent elevated risks.

 The author, himself, admits that this may not be resolved without randomized control trials (with some diagnosed insomniacs not receiving hypnotics or receiving non-drug therapies instead). However, it may be difficult to obtain ethics approval for a study allocating insomnia sufferers to a non-treatment control over a long period of time (e.g 2-3 years to measure long term health outcomes). The only opportunity might be to compare pharmacotherapy alone with cognitive/behavior therapy with long term follow-ups of sleep and many health outcomes. This should be acceptable ethically.

 The difficulty with drawing causal conclusions from epidemiological studies is nicely illustrated by one of the cited references with some of the most compelling evidence, the study by Anderson, *et al* ^1^. In their study they reported on an insomnia subset of 5,773 of which 75% were treated with a prescription medication and the other 25% were not and all were followed up after a 12 month period. The treated subset was more likely to have a mental health diagnosis and anti-depressant medications. These differences were significant between the groups at baseline which supports the notion that it is the higher morbidity patients more likely to receive hypnotic medication. These initial morbidity differences were then amplified at the follow-up and the medicated group showed greater increases in health costs ($4276 vs $2309) over that time. This is strong circumstantial evidence that the hypnotic medications were causing an increase in medical costs. At least one could say with greater certainty that the medications were not curing their insomnia and reducing health costs. The only lingering doubt, however, is not knowing the outcome had they not been medicated. Would they have been even worse off, no different, or, as Dr. Kripke is suggesting, better off? We simply cannot be certain until randomized controlled trials are conducted.

 A crude but good analogy to the point I am making is the effect of hospitalization. Should a 70 year old with a severe chest infection be hospitalized? Those admitted to hospital certainly have a higher morbidity and mortality risk. Does that mean someone with these symptoms will be better advised to stay out of the hospital?

 In the mean time, the weight of the evidence presented by the author is compelling. My only suggestion is for the author not to slip into the use of terminology that the causal connection is proven. By doing so he is in jeopardy of reducing the strength and consistency of the correlation data. Let the data be presented and just percolate in the reader's mind. It will thus be more effectively taken up than if the author takes the one step too far to insist upon the causal link. Already there is too much research being presented implying causal links between sleep variables (e.g. reported total sleep time) and health outcomes from epidemiological studies. Let's not contribute to this inappropriate trend.

I have read this submission. I believe that I have an appropriate level of expertise to confirm that it is of an acceptable scientific standard, however I have significant reservations, as outlined above.

## References

[1] AndersonLHWhitebirdRRSchultzJMcEvoyCEKreitzerMJGrossCR: Healthcare utilization and costs in persons with insomnia in a managed care population. *Am J Manag Care*.2014;20(5) :e157-65 25326930

# Response

**Published:** 2017-06-21

**Authors:** Daniel F. Kripke

**Affiliations:** University of California, San Diego, USA

Dr. Lack’s very thoughtful emphasis on the problem of causality highlights a crucial issue for readers of this manuscript about hypnotic costs.  His review contributes to the discussion already in the manuscript calling for more studies including studies of causality.  Likewise, the manuscript specifically acknowledged the problem of associational studies potentially being confounded by comorbidities.  The problem of comorbidities is emphasized a bit more in Version 2.  Until more reliable data become available, the manuscript argues that managed care and insurance administrators should weigh the best available evidence of whether the costs of hypnotic harms are likely to exceed the value of hypnotic benefits.

 As in most areas of medicine, timely decisions about hypnotic treatments and reimbursements must be made based on incomplete and imperfect evidence.  Decisions need to be made daily and cannot be deferred for years more study. We still do not have definitive randomized controlled trials about whether cigarettes cause cancer in humans.  After millions of people had died from cancers “associated with” cigarette smoking, it became time to advise against cigarette smoking based on available evidence that association probably indicates a large element of causation. 

 The reader may be sure that this manuscript on costs never implies causation without causal evidence, but uses the word “association” whenever there is insufficient evidence of an element of causation.  Some added emphasis on this distinction is included in the Version 2 revision.

 This response to both reviews is a welcome opportunity to elaborate on the issues of causation versus association that were not presented at length in a manuscript about costs, since that would repeat much detail previously published. ^1,2^  However, just as it would be an error to slip into language implying causation when only association has been demonstrated, it would be an error not to recognize existing human trials and meta-analysis confirming evidence of causation.  Moreover, in the case of hypnotics, as with cigarettes, there is much animal causal data and *in vitro* data supporting the evidence of association studies.  The sleep disorders community has been slow to recognize and replicate the existing findings as the causal evidence of serious hypnotic risks has accumulated.   

 Joya et al. ^3^ performed formal meta-analysis of 38 randomized controlled trials of 8828 participants randomized to 4 hypnotics and 4383 participants randomized to placebos, in whom the overall infection causation hazard ratio was 1.44 (1.25-1.64, confidence interval, P<0.00001).  If any manufacturer could do a meta-analysis showing that this analysis was wrong, would they not have done so?  How much more evidence is needed?  As a matter of fact, Sanofi subsequently did an independent analysis of their Ambien data and informed the FDA that they could confirm that Ambien (zolpidem CR) causation of infections such as influenza was “probably real.” ^4^ The FDA expert agreed. Would skeptics nevertheless question that hypnotic causation of infections such as influenza creates medical costs and sometimes deaths?

 For an invited lecture, Kripke did an informal analysis of incident depressions in randomized trials of 4 hypnotics (including data available through the FDA that manufacturers did not publish). There were 5535 participants randomized to hypnotics with 2% incidence of depression and 2318 participants randomized to placebos with 0.9% incidence, yielding a depression causation risk ratio of 2.1 (P<0.002). ^5^  If any manufacturer could do a larger, more formal meta-analysis showing that hypnotics do not cause depression, would one not think they would have published?  In an important study of 593 participants randomized to eszopiclone and 195 randomized to placebo, the rate of discontinuation for depression was 2.0% in the eszopiclone group and 0.0% in the placebo group, a result that appears statistically significant.  In their modesty, the authors did not even mention that the study had proven that eszopiclone causes infection. ^6^  Note that these were new, incident depressions, not exacerbations, since the study excluded patients with any DSM-IV Axis I psychiatric diagnosis at baseline. ^6^ Would skeptics nevertheless question that depression elevates medical costs and sometimes causes suicides?

 The previous detailed review also discussed at length the many death certificates that mention hypnotics as one of the causes of death and likewise discussed why these are probably underestimates. ^1^  For 2015, the U.S. CDC’s WONDER data base listed over 10,000 deaths with causes of death that included a barbiturate, benzodiazepine, or a Z-drug (the Z-drug category included anti-epileptics), but for many reasons, the 10,000 reported deaths is believed to be an underestimate.  Would skeptics nevertheless argue that death certificates listing a hypnotic as a cause of death are evidence for association, not causality?

 To summarize, there are certainly adequate data confirming that hypnotics cause some of the harms discussed in this manuscript and some of costs resulting. There are also controlled trials showing that hypnotics cause impaired balance and poor on-road driving performance, though controlled trials with automobile accidents or falls as endpoints have not yet reached adequate size. Further research is needed to better define the magnitude of costs from hypnotic harms, but some causal role of hypnotics in several of the harms resulting has already been confirmed. 

 Dr. Lack’s comments are very helpful in reiterating the need for expanded studies.  The problems of ethical approval are not insurmountable. If it is ethical to administer hypnotics to patients in short-term hospitalization, would it not be ethical to perform randomized trials focusing on whether hypnotic prescribing increases or decreases hospital costs?  Short-term hospital studies might also provide useful evidence regarding causality of serious infections and depression.  Even for early death as an endpoint, short-term randomizing clinical trials could be ethical and have adequate power.  Since the epidemiology suggests that the greatest risk-ratio of hypnotics may be within the first 30 administrations, mainly among frail and elderly patients, randomized placebo-controlled clinical trials might only need to last 30 days among frail and vulnerable participants (who currently receive much of the hypnotics prescribed). To be sure, large numbers of participants would be needed for adequate power in relatively brief randomization designs.  Another alternative would be the randomized education-patient-empowerment technique initiated by Dr. Tannenbaum. ^7^ Another alternative would be Mendelian randomization studies.  Randomizing studies of causality are feasible if those who advocate and provide hypnotics recognize their responsibilities to assess the risks and costs.

 1.  Kripke DF. Hypnotic drug risks of mortality, infection, depression, and cancer: but lack of benefit [version 2]. *F1000Research*. 2017; **5**: 918 ( http://dx.doi.org/10.12688/f1000research.8729.2 ).

 2.  Kripke DF. Mortality risk of hypnotics:  strengths and limits of evidence. *Drug Saf*. 2016; **39**: 93-107.

 3.  Joya FL, Kripke DF, Loving RT, Dawson A, Kline LE. Meta-analyses of hypnotics and infections: eszopiclone, ramelteon, zaleplon, and zolpidem. *J Clin Sleep Med*. 2009; **5**(4): 377-83.

 4.  Farkas R. Center for Drug Evaluation and Research Approval Package for:  Application Number: 019908Orig1s032s034  021774Orig1s013s015.  Silver Spring, MD, FDA. Accessed 2013:  accessible through https://www.accessdata.fda.gov/scripts/cder/daf/ under zolpidem.

 5.  Kripke DF. Greater incidence of depression with hypnotics than with placebo. *BMC Psychiatry*. 2007; **7:42**.

 6.  Krystal AD, Walsh JK, Laska E, Caron J, Amato DA, Wessel TC, Roth T. Sustained efficacy of eszopiclone over 6 months of nightly treatment:  results of a randomized, double-blind, placebo-controlled study in adults with chronic insomnia. *Sleep*. 2003; **26**(7): 793-9.

 7.  Tannenbaum C, Martin P, Tamblyn R, Benedetti A, Ahmed S. Reduction of inappropriate benzodiazepine prescriptions among older adults through direct patient education: the EMPOWER cluster randomized trial. *JAMA Intern Med*. 2014; **174**(6): 890-8.

# Peer review: Referee response for version 1

**DOI:** 10.5256/f1000research.12228.r22745

**Published:** 2017-05-25

**Authors:** Michael A. Grandner

**Affiliations:** 1 Sleep and Health Research Program, Department of Psychiatry, College of Medicine, University of Arizona, Tucson, AZ, USA

Overall this is an important contribution. A few relatively minor concerns:

 1. The term "proven" is used quite a bit. It may be better to use the more qualified "shown" or "demonstrated."

 2. The paper by Moloney and colleagues (2011) demonstrates quite clearly that rates of hypnotic use are high, not really tied to insomnia diagnosis, and often chronic. This may aid in making the case in this article.

 3. In addition to mentioning the ACP guideline, even the recent AASM guidelines for prescription hypnotic use state that evidence for using hypnotics is overall low.

 4. There is often causal language that may be more strong than the evidence warrants. For example, it is not clear that the role of hypnotics in suicides is causal. Certainly, anecdotal evidence suggests that people overdose on sleeping pills, but the cited report has a whole paragraph about suicides that makes mostly circumstantial connections. Clearly there is a relationship, but the causal role of hypnotics is not so clearly demonstrated.

 5. If hypnotics don't produce benefits, then why do people keep taking them? For example, even if the medications do not directly impact on sleep, there may be evidence that the effects on suppressing memory may outweigh the effects on sleep. So they may not actually improve sleep much but reduce the memory or perception of sleeplessness. If this reduces the discomfort associated with insomnia (i.e., it removes your memory for the events) then it could potentially have some sort of reinforcing benefit in that regard. Just saying that there are no benefits may be a bit oversold.

 6. The "Detailed estimates" section does not actually include detailed estimates. Rather, it provides several general estimates for a number of specific pathways by which hypnotics could induce harms. Perhaps this section could be better labeled.

I have read this submission. I believe that I have an appropriate level of expertise to confirm that it is of an acceptable scientific standard, however I have significant reservations, as outlined above.

# Response

**Published:** 2017-05-26

**Authors:** Daniel F. Kripke

**Affiliations:** University of California, San Diego, USA

Dr. Grandner’s has provided several very helpful suggestions which will be incorporated in a revision of the manuscript after the initial reviews.  He mentioned two points of interest deserving comment here.

         First, there is the question of whether there is evidence that hypnotics cause suicide, going beyond the extensive evidence for association.  This is discussed in more detail in my recent more detailed review of the evidence for hypnotic harms:  doi: 10.12688/f1000research.8729.2 .  There are several kinds of evidence.  Controlled trials and meta-analysis have shown that hypnotics cause incident depressions and cause incident infections.  Controlled trials are our best tests of causality.  There is much new evidence that infection causes depression and suicides, and depression itself is widely believed to be the main cause of suicide.  Second, in a narrower sense, when a medical examiner reports a hypnotic drug as a cause of intentional overdose death (and there are thousands of such examples), that is an expert finding of causality.  Since no specific costs of suicides and suicide attempts could be included in the hypnotic cost estimates offered, causality of suicide is not a key issue for the manuscript being reviewed.  More study is certainly needed.  Incidentally, my petition to the FDA specifically mentioned suicides as one endpoint which should be incorporated in expanded Phase IV randomized safety trials of hypnotics.

            Second, there is the question of why people keep taking hypnotics.  This is a question for the ages, since throughout the history of magical beliefs and shamanism, people have consumed countless remedies including sleep remedies that are scientifically considered non-beneficial.  Moreover, benzodiazepine-agonist hypnotics are known to be addicting drugs that are sometimes sought for pleasure, but equally important, these hypnotics cause withdrawal insomnia.  There is much evidence that people persist in using hypnotics because of the withdrawal insomnia and anxiety that occur when consumers try to stop.  As the reviewer suggested, people may take hypnotics seeking the amnesia that hypnotics produce, just as alcoholics drown their sorrows.  That amnesia has not been recognized and advertised as a medical benefit.  I am not aware of evidence that hypnotic-induced amnesia is medically beneficial, though it does often elicit a favorable subjective report.  In hospitals, there might be an argument for helping patients to forget their anxieties, pains, and discomforts, but if the same amnesic effects contribute to falls, confusion, and dementia, it may be a costly strategy.
